# Effect of sacubitril/valsartan on brain natriuretic peptide level and prognosis of acute cerebral infarction

**DOI:** 10.1371/journal.pone.0291754

**Published:** 2023-09-21

**Authors:** Xiaozhu Shen, Chen Gong, Mengqian Liu, Yi Jiang, Yiwen Xu, Zhonglin Ge, Zhonghai Tao, Nan Dong, Juan Liao, Liqiang Yu, Qi Fang

**Affiliations:** 1 Department of Geriatrics, Lianyungang Hospital, Affiliated to Jiangsu University (Lianyungang Second People’s Hospital), Lianyungang, China; 2 Bengbu Medical College, Bengbu, China; 3 Department of Infectious Diseases, The First Affiliated Hospital of Ningbo University, Ningbo, China; 4 Department of Neurology, Lianyungang Second People’s Hospital, Lianyungang, China; 5 Department of Neurology, Suzhou Industrial Park Xinghai Hospital, Suzhou, China; 6 Department of Neurology, First Affiliated Hospital of Soochow University, Suzhou, China; Public Library of Science, UNITED KINGDOM

## Abstract

**Background and purpose:**

Previous studies demonstrated that elevated brain natriuretic peptide (BNP) level is associated with adverse clinical outcomes of acute cerebral infarction (ACI). Researchers hypothesized that BNP might be a potential neuroprotective factor against cerebral ischemia because of the antagonistic effect of the natriuretic peptide system on the renin-angiotensin system and regulation of cardiovascular homeostasis. However, whether decreasing the BNP level can improve the prognosis of ACI has not been studied yet. The main effect of sacubitril/valsartan is to enhance the natriuretic peptide system. We investigated whether the intervention of plasma BNP levels with sacubitril/valsartan could improve the prognosis of patients with ACI.

**Methods:**

In a randomized, controlled, parallel-group trial of patients with ACI within 48 hours of symptom onset and need for antihypertensive therapy, patients have randomized within 24 hours to sacubitril/valsartan 200mg once daily (the intervention group) or to conventional medical medication (the control group). The primary outcome was a change in plasma BNP levels before and after sacubitril/valsartan administration. The secondary outcomes included plasma levels of brain-derived neurotrophic factor (BDNF), Corin and neprilysin (NEP) before and after medication, the modified Rankin scale, and the National Institutes of Health Stroke Scale (at onset, at discharge, 30 days, and 90 days after discharge).

**Results:**

We evaluated 80 eligible patients admitted to the Stroke Center of Lianyungang Second People’s Hospital between 1st May, 2021 and 31st June, 2022. Except for 28 patients excluded before randomization and 14 patients who did not meet the criteria or dropped out or lost to follow-up during the trial, the remaining 38 patients (intervention group: 17, control group: 21) had well-balanced baseline features. In this trial, we found that plasma BNP levels (*P* = 0.003) decreased and NEP levels (*P* = 0.006) increased in enrolled patients after treatment with sacubitril/valsartan. There were no differences in plasma BDNF and Corin levels between the two groups. Furthermore, no difference in functional prognosis was observed between the two groups (all *P* values>0.05).

**Conclusions:**

Sacubitril/valsartan reduced endogenous plasma BNP levels in patients with ACI and did not affect their short-term prognosis.

## Introduction

According to previous research, brain natriuretic peptide (BNP) is a sensitive indicator of cardiac dysfunction released by cardiomyocytes after mechanical traction stress in the ventricular wall. Other parts of the body also secrete BNP and express its receptors, such as the telencephalon, Cerebral cortex, Amygdaloid nuclei, Olfactory bulb, Striatum, Hypothalamus, etc [1]. In addition to cardiovascular, the effects of BNP on cerebrovascular vessels are increasingly studied. BNP has been extensively studied as a predictor of outcome in acute cerebral infarction (ACI) [2, 3]. Increased plasma BNP (>100pg/mL) levels were independently associated with higher in-hospital mortality, and patients with higher BNP levels tend to have unfavorable 90-day outcomes (mRS ≥3) [4]. High BNP levels suggest that the short-term prognosis of stroke is also not optimistic [5]. BNP levels were inversely correlated with favorable outcomes in stroke patients at discharge [6, 7]. Studies have proposed that BNP might function as a potential neuroprotective factor against cerebral ischemia based on the antagonistic effect of the natriuretic peptide system on the renin-angiotensin system and the regulation of cardiovascular homeostasis [8]. Cerebroventricular injection of type A natriuretic peptide (ANP) in acute ischemic stroke model rats also observed significant improvements in neurological function, infarct volume, and edema [9]. The same type of C natriuretic peptide (CNP) also has an endogenous neuroprotective effect on neuronal survival after mouse cerebellar ischemic brain injury, reducing infarct size and improving neural function [10]. Unfortunately, whether the intervention of plasma BNP levels in humans can alter the clinical outcome of ACI patients has not been studied yet. Sacubitril/valsartan, which has been widely used in clinical practice in recent years, is a combination of a neprilysin inhibitor (sacubitril) and an angiotensin receptor blocker (valsartan) [11]. It works by both enhancing the natriuretic peptide system by inhibiting neprilysin (NEP) and inhibiting the renin-angiotensin-aldosterone system (RAAS) by blocking angiotensin II receptors. Therefore, it can be concluded that sacubitril/valsartan can inhibit both the RAAS system and NEP to reduce the already elevated BNP levels and enhance the protective effects of endogenous natriuretic peptides [12] on target organs. We designed this study to determine whether sacubitril/valsartan could improve the clinical outcomes of ACI patients by enhancing the action of the endogenous natriuretic peptide system, which is the primary benefit of the drug combination.

## Methods

### Study design and population

Our study was a randomized, controlled, parallel-group trial conducted at the Stroke Center of Lianyungang Second People’s Hospital. From 1st May 2021 to 31st June 2022, 80 patients aged ≥18 years who had ACI confirmed by computed tomography (CT) or magnetic resonance imaging (MRI) of the brain within 48 hours of symptom onset and required antihypertensive therapy (Real-time systolic BP ≥180mmHg or diastolic BP≥100mmHg, or patients with severe cardiac insufficiency, aortic dissection, hypertensive encephalopathy, or having a history of hypertension and regularly taking anti-hypertensive medication) were consecutively enrolled. Patients who fulfilled one of the following conditions were excluded from the trial: (1) Patients with cerebral hemorrhage and occupancy (emergency head CT excludes cerebral hemorrhage, while post-infarction hemorrhage is not excluded if the patient was hospitalized); (2) Patients with transient ischemic attack; (3) Complicated with severe infection or septic shock; (4) Combined with hypotension or hyperkalemia; (5) Obvious hepatic and renal insufficiency (glomerular filtration rate <30ml/min or Child-Pugh class C); (6) Endocrine, immune, or neoplastic diseases; (7) History of severe trauma with surgical treatment within 30 days; (8) Presence of blindness, deafness or communication disorders; (9) Diagnosed as schizophrenia, affective disorder, organic mental disorder or mental retardation; (10) had a contraindication to sacubitril/valsartan; (11) Pregnancy. All patients provided written informed consent. This trial received ethical approval from the ethics committees of Lianyungang Second People’s Hospital (No.2020010), and it was registered in the Chinese Clinical Trials Registry (ChiCTR2100047408).

### Data collection and assessment

At the time of enrollment, information on demographic factors, clinical characteristics, and medical history was gathered. This information included age, gender, body mass index, history of atrial fibrillation (previous atrial fibrillation and new-onset atrial fibrillation at stroke), hypertension, diabetes, prior TIA or stroke, statin use, current smoking (any usage of cigarette per day in the past 30 days) and drinking (drinking more than 100ml (alcohol content>50%) per day on average and drinking for more than 1 year; abstaining from drinking for more than 1 year is not).

With the consent of potentially eligible patients, venous blood was extracted from patients at admission and immediately sent for examination. Laboratory tests included; white blood cell count, red blood cell count, hemoglobin, blood platelet count, neutrophil count, red cell distribution width, alanine aminotransferase, aspartate transaminase, free thyroxine, free triiodothyronine, thyroid-stimulating hormone, glucose, glycated hemoglobin A1c, blood urea nitrogen, creatinine, uric acid, total cholesterol, triglycerides, low-density lipoprotein cholesterol, high-density lipoprotein cholesterol, international normalized ratio, prothrombin time, activated partial thromboplastin time, D2-dimer, fibrinogen, troponin and C-reactive protein.

Ultrasonic cardiograph findings included left atrium diameter, left ventricular end-diastolic diameter, and left ventricular ejection fraction. Trial of Org 10172 in acute stroke treatment (TOAST) classification was divided into large-artery atherosclerosis (LAA), cardioembolic (CE), and others. LAA was defined as >50% stenosis of the vessel lumen in the extracranial or intracranial segment of the internal carotid artery, M1/M2 segment of the middle cerebral artery, or anterior cerebral artery [13]. The severity and prognosis of ACI were assessed by two trained neurologists using the National Institutes of Health Stroke Scale (NIHSS) and the modified Rankin Scale (mRS) at onset, at discharge (averaging around 14 days), and at 30 and 90 days after discharge.

### Plasma soluble BDNF, Corin, BNP and NEP levels tests

Plasma-soluble brain-derived neurotrophic factor (BDNF) level was measured using a human BDNF ELISA kit (Catalog: JL11683, Jianglai Inc., Shanghai, China); plasma-soluble Corin level was measured using a human Corin ELISA kit (Catalog: IC-Corin-Hu, IC ImmunoClone Inc., Shanghai, China); plasma-soluble NEP was measured using a human NEP ELISA kit (Catalog: JL15469, Jianglai Inc., Shanghai, China); clinical data were used to determine the plasma-soluble BNP level. Among them, our primary outcome was a change in plasma BNP levels before and after administration of sacubitril/valsartan. While other measures, including plasma levels of BDNF, Corin, and NEP before and after medication, were secondary outcomes of our analysis.

### Blood pressure monitoring

The blood pressure (BP) was measured with the participant in a supine position using a standard mercury sphygmomanometer by a trained nurse. After randomization, the BP was measured at 7 a.m. and 3 p.m. every day for a week until hospital discharge or death.javascript:void(0); Systolic blood pressure fluctuation (SBPF) was calculated using the following equation: SBPF = {(SBP_1,max_-SBP_1,min_)+(SBP_2,max_-SBP_2,min_)+…+(SBP_n,max_-SBP_n,min_)}/n, where ‘SBP_n,max_’ was the maximum systolic BP on day ‘n’ of hospitalization, ‘SBP_n,min_’ was the minimum systolic BP on day ‘n’ of hospitalization.

### Randomization and treatments

Participants were allocated in a 1:1 ratio to receive either sacubitril/valsartan treatment (intervention group) or conventional medical therapy (control group). The randomization sequence was computer-generated. A specialist who was not involved in the trial performed the randomization process. Sacubitril/valsartan 200mg once daily or conventional medical drugs (Valsartan 160mg once daily) were given as soon as possible within 24 hours of randomization, and other post-admission treatment regimens remained as consistent as possible. Over the course of more than 3 months, sacubitril/valsartan has been given.

### Management of blood samples

5ml peripheral venous blood samples were collected from subjects before and 5 days after administration and stored in EDTA anticoagulation tubes. 2ml of plasma was separated by centrifugation at 5°C for 60μl and stored in a refrigerator at -80°C to await plasma proteins.

### Statistical analysis

All data were analyzed by using the SPSS software (IBM SPSS Statistics for Windows, version 26.0; IBM Corp, Armonk, NY, USA) and GraphPad Software (GraphPad Prism for Windows, version 9.0.0; San Diego, California USA). Kolmogorov-Smirnov test was used to assess the normality of continuous variables. The normative continuous variables were presented as mean ± standard deviation (SD). Median and interquartile range (IQR) were used to describe continuous variables if non-normal distributed. Differences in baseline data were compared using Student’s t-test or Mann-Whitney U test for continuous variables and Fisher exact test for categorical variables. For those indicators of pre- and post-administration testing, such as BDNF, NEP, BNP, and Corin, Wilcoxon sign-rank tests were performed to assess differences. Two-way repeated measures ANOVA were used to analyze patients’ blood pressure monitoring status. Missing values were handled using a pairwise deletion method. A 2-tailed *P* value<0.05 was considered statistically significant.

## Results

### Study population and clinical characteristics

**Fig 1:**
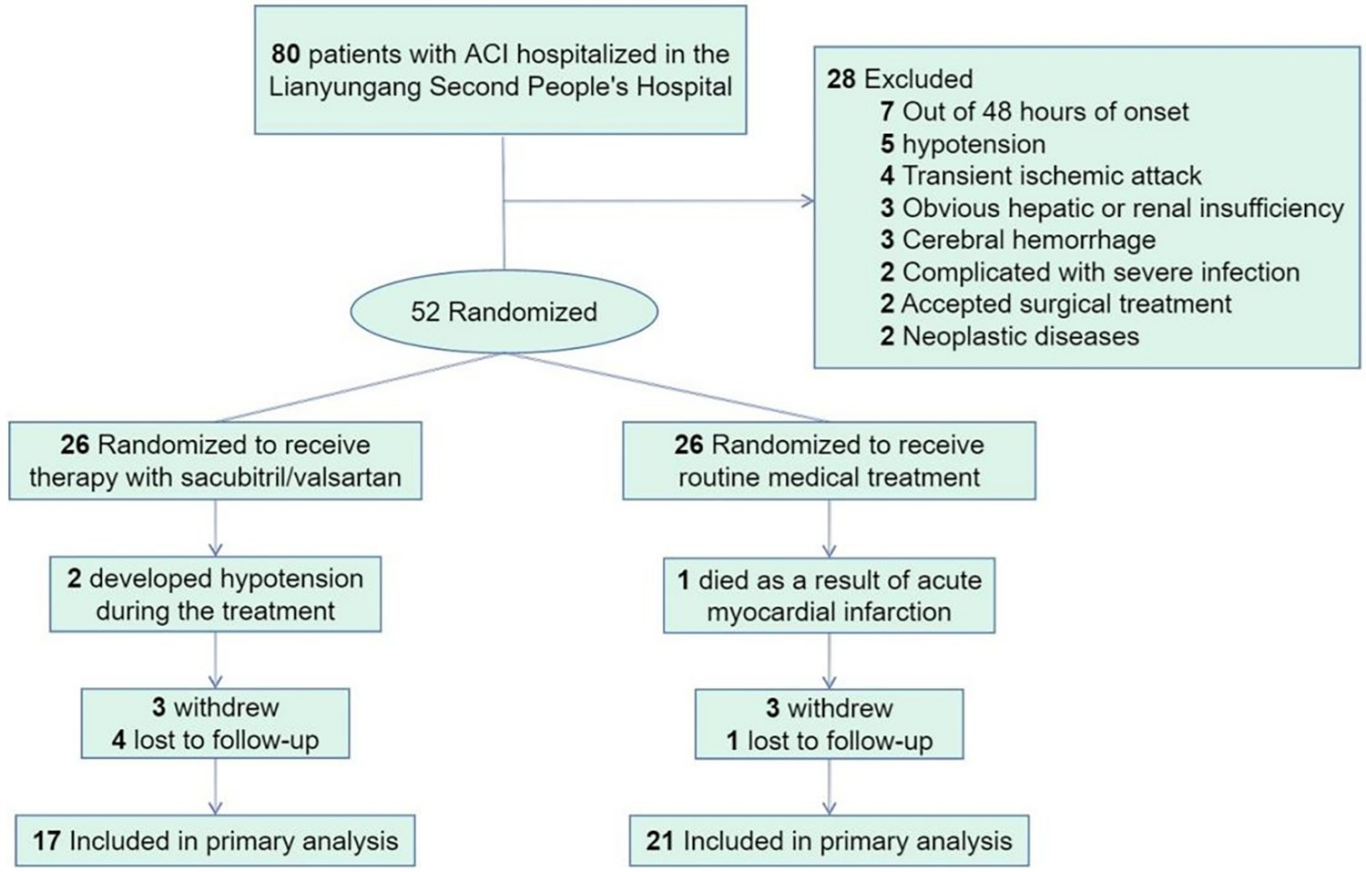
Flow diagram of included and excluded patients. ACI, acute cerebral infarction.

There were 80 patients with ACI hospitalized in the Lianyungang Second People’s Hospital screened and met the eligibility criteria between 1st May 2021 and 31st June 2022 enrolled in this study (Fig 1). The remaining 52 were divided equally between the intervention group and the control group by random assignment after 28 of them were eliminated based on the exclusion criteria (26 to each group). During 90 days of follow-up visits, 6 people dropped out (intervention group: 3, control group: 3) and 5 people lost contact (intervention group: 4, control group: 1). Furthermore, 2 people in the intervention group developed severe hypotension which could not be sustained during the trial and 1 person in the control group died of acute myocardial infarction so complete data were not available, leaving only 38 people available for analysis.

Table 1 shows demographic, clinical, and laboratory characteristics of randomized participants at baseline. Baseline characteristics of patients were well balanced, and the mean age was 72.45 years (SD, 11.63), 52.6% were women, and 92.1% were LAA subtypes.

**Table 1:** Baseline characteristics of the participants.

Parameter	Overall (N = 38)	Intervention group (N = 17)	Control group (N = 21)	*P* value
Age, Mean (SD)−year	72.45±11.63	73.65±10.47	71.48±12.66	0.574^a^
Sex−no. (%)				0.328^c^
Male	18 (47.4%)	10 (58.8%)	8 (38.1%)	
Female	20 (52.6%)	7 (41.2%)	13 (61.9%)	
BMI, Mean (SD)−kg/m^2^	24.97±3.45	25.19±3.40	24.80±3.56	0.736^a^
**Medical history**				
Atrial fibrillation−no. (%)	5 (13.2%)	2 (11.8%)	3 (14.3%)	0.999^c^
Hypertension−no. (%)	31 (81.6%)	16 (94.1%)	15 (71.4%)	0.104^c^
Diabetes mellitus−no. (%)	17 (44.7%)	7 (41.2%)	10 (47.6%)	0.752^c^
History of TIA or stroke−no. (%)	16 (42.1%)	7 (41.2%)	9 (42.9%)	0.999^c^
History of smoking−no. (%)	9 (23.7%)	3 (17.6%)	6 (28.6%)	0.476^c^
History of drinking−no. (%)	6 (15.8%)	2 (11.8%)	4 (19.0%)	0.672^c^
History of statin use−no. (%)	16 (42.1%)	10 (58.8%)	6 (28.6%)	0.099^c^
**Biochemical variables**				
WBC, Mean (SD)−10^9/L	7.15±2.61	6.85±1.85	7.39±3.12	0.535^a^
RBC, Mean (SD)−10^12/L	4.64±0.94	4.94±1.07	4.39±0.76	0.071^a^
Hb, Mean (SD)−g/L	134.53±28.43	140.06±17.55	130.05±34.67	0.287^a^
PLT, Mean (SD)−10^9/L	230.68±68.65	233.35±76.16	228.52±63.77	0.833^a^
NEUT, Median (IQR)−10^9/L	4.75 (3.51,6.42)	4.76 (2.90,6.58)	4.73 (3.70,6.96)	0.547^b^
RDW, Mean (SD)−%	12.91±1.36	13.20±1.75	12.68±0.93	0.244^a^
ALT, Mean (SD)−U/L	20.82±8.48	21.47±7.10	20.29±9.59	0.674^a^
AST, Mean (SD)−U/L	22.32±5.39	22.94±4.99	21.81±5.77	0.528^a^
FT4, Mean (SD)−pmol/L	12.22±2.18	11.45±2.03	12.84±2.15	0.049^a^
FT3, Mean (SD)−pmol/L	4.74±0.75	4.90±0.75	4.61±0.74	0.235^a^
TSH, Mean (SD)−μIU/mL	1.73±1.10	1.54±1.05	1.89±1.13	0.336^a^
Glucose, Median (IQR)−mmol/L	6.71 (6.28,9.23)	6.77 (6.20,9.80)	6.50 (6.27,9.25)	0.941^b^
HbA1c, Median (IQR)−%	6.25 (5.88,8.20)	6.00 (5.80,8.70)	6.50 (5.95,8.00)	0.453^b^
BUN, Median (IQR)−mmol/L	6.30 (4.90,8.88)	5.70 (4.70,8.80)	6.80 (5.00,8.95)	0.557^b^
Cr, Median (IQR)−μmol/L	71.00 (50.00,104.75)	61.00 (50.00,94.00)	74.00 (51.50,109.50)	0.587^b^
UA, Median (IQR)−μmol/L	314.00 (261.50,400.50)	347.00 (252.00,439.50)	309.00 (277.00,386.50)	0.953^b^
TC, Median (IQR)−mmol/L	4.62 (4.14,5.63)	4.93 (4.16,5.66)	4.38 (3.74,5.53)	0.419^b^
TG, Median (IQR)−mmol/L	1.83 (1.07,2.74)	1.85 (1.19,3.30)	1.66 (1.07,2.07)	0.481^b^
LDL-C, Median (IQR)−mmol/L	2.96 (2.29,3.56)	2.99 (2.43,3.76)	2.92 (2.18,3.57)	0.597^b^
HDL-C, Median (IQR)−mmol/L	1.14 (0.95,1.40)	1.20 (0.97,1.37)	1.10 (0.92,1.43)	0.618^b^
INR, Median (IQR)	1.04 (0.98,1.09)	1.02 (0.95,1.05)	1.08 (0.99,1.13)	0.015^b^
PT, Median (IQR)−s	11.80 (11.30,12.23)	12.00 (11.20,12.35)	11.80 (11.45,12.15)	0.735^b^
APTT, Median (IQR)−s	30.45 (29.30,32.00)	29.90 (28.75,32.10)	31.20 (29.70,31.85)	0.311^b^
D2-dimer, Median (IQR)−ng/mL	168.00 (100.25,263.00)	130.00 (97.50,269.50)	182.00 (114.50,264.00)	0.419^b^
FIB, Median (IQR)−g/L	4.25 (3.72,4.81)	4.12 (3.59,4.44)	4.70 (3.85,5.14)	0.086^b^
TnI, Median (IQR)−pg/mL	5.10 (3.60,9.70)	5.10 (3.15,8.85)	4.70 (3.90,14.35)	0.692^b^
CRP, Median (IQR)−mg/L	3.23 (1.13,12.35)	3.05 (0.96,11.28)	3.40 (1.14,12.45)	0.895^b^
**Ultrasonic cardiograph variables**				
LAD, Median (IQR)−mm	36.00 (33.75,38.00)	36.00 (33.50,39.00)	35.00 (33.50,37.50)	0.836^b^
LVEDD, Median (IQR)−mm	46.00 (43.00,47.25)	47.00 (43.50,48.00)	45.00 (43.00,47.00)	0.254^b^
LVEF, Median (IQR)−mm	61.00 (58.00,64.00)	62.00 (59.50,63.00)	60.00 (55.50,64.00)	0.452^b^
**NIHSS**, Median (IQR)	9.00 (3.00.15.00)	4.00 (3.00,14.50)	10.00 (3.00,15.00)	0.367^b^
**TOAST−no. (%)**				0.999^c^
LAA	36 (92.1%)	16 (94.1%)	19 (90.5%)	
CE	3 (7.9%)	1 (5.9%)	2 (9.5%)	

Abbreviations: BMI, body mass index; WBC, white blood cell count; RBC, red blood cell count; Hb, hemoglobin; PLT, blood platelet count; NEUT, neutrophil count; RDW, red cell distribution width; ALT, alanine aminotransferase; AST, aspartate transaminase; FT4, free thyroxine; FT3, free triiodothyronine; TSH, thyroid-stimulating hormone; HbA1c, glycated hemoglobin A1c; BUN, blood urea nitrogen; Cr, creatinine; UA, uric acid; TC, total cholesterol; TG, total glyceride; LDL-C, low-density lipoprotein cholesterol; HDL-C, high-density lipoprotein cholesterol; INR, international normalized ratio; PT, prothrombin time; APTT, activated partial thromboplastin time; FIB, fibrinogen; TnI, troponin I; CRP, C-reactive protein; LAD, left atrium diameter; LVEDD, left ventricular end-diastolic diameter; LVEF, left ventricular ejection fraction; NIHSS, National Institutes of Health Stroke Scale; TOAST, Trial of Org 10172 in acute stroke treatment; LAA, large-artery atherosclerosis; CE, cardioembolic.

^a^ analyzed by Student’s t-test

^b^ analyzed by Mann-Whitney U test

^c^ analyzed by Fisher exact test.

### BDNF, NEP, BNP and Corin levels

**Fig 2:**
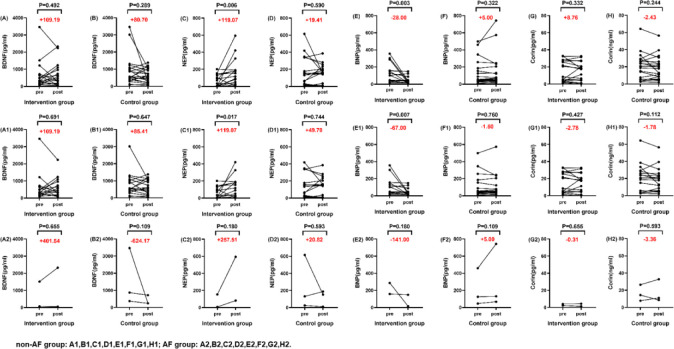
Comparison of indicators before and after treatment. BDNF, brain-derived neurotrophic factor; NEP, neprilysin; BNP, brain natriuretic peptide. Note: all *P* values were obtained using the Mann-Whitney U test.

Mann-Whitney U test was used to analyze the baseline levels of each test indicator (Table 2). As we can see, there was a difference in the baseline levels of NEP before medication between the two groups (*P* = 0.042), while there was no statistical difference in the baseline levels of other indicators, including BDNF, BNP, and Corin (all *P* values>0.05). Wilcoxon sign-rank test was used to evaluate the changes in indicators before and after medication (Table 3). Patients randomized to sacubitril/valsartan achieved more elevated BDNF and NEP levels compared with the control group, and NEP level was significantly higher after treatment than before (*P* = 0.006). BNP levels decreased in the intervention group after treatment with sacubitril/valsartan (*P* = 0.003), while the slight increase in BNP levels in the control group was not statistically significant (*P* = 0.322). Corin levels were slightly elevated in the intervention group of patients receiving sacubitril/valsartan but decreased in the control group. In the subgroup analysis between the AF group and the non-AF group, we found that the overall difference was consistent with the difference in the non-AF group, that is, the NEP level was significantly higher in the non-AF group after treatment than before treatment(*P* = 0.017), and the intervention group BNP levels decreased after sacubitril/valsartan treatment (*P* = 0.007) (Fig 2).

**Table 2:** Baseline levels of indicators before medication.

Parameter	Overall (N = 38)	Intervention group (N = 17)	Control group (N = 21)	*P* value
BDNF, Median (IQR) pg/mL	499.71 (283.78, 952.27)	339.44 (176.18,681.30)	528.34 (395.26,1051.71)	0.086
BNP, Median (IQR) pg/mL	56.00 (41.25,164.75)	103.00 (45.00,222.50)	51.00 (35.50,169.50)	0.340
NEP, Median (IQR) pg/mL	67.41 (15.47,153.23)	34.84 (0.00,136.55)	130.77 (23.20,257.63)	0.042
Corin, Median (IQR) ng/mL	19.54 (4.92,27.00)	8.58 (3.58,27.26)	20.34 (5.65,27.47)	0.297

Abbreviations: BDNF, brain-derived neurotrophic factor; BNP, brain natriuretic peptide; NEP, neprilysin.

**Table 3:** Changes of indicators before and after medication.

Parameter	Overall (N = 38)			Intervention group (N = 17)			Control group (N = 21)		
	Before medication	After medication	*P* value	Before medication	After medication	*P* value	Before medication	After medication	*P* value
BDNF,Median (IQR) pg/mL	499.71 (283.78,952.27)	550.21 (230.37,1034.08)	0.744	339.44 (176.18,681.30)	448.63 (176.61,1025.83)	0.492	528.34 (395.26,1051.71)	609.04 (241.74,1046.72)	0.289
BNP, Median (IQR) pg/mL	56 (41.25,164.75)	45.5 (27,124)	0.114	103 (45,222.5)	35 (15,104.5)	0.003	51 (35.5,169.5)	56 (41,167.5)	0.322
NEP, Median (IQR) pg/mL	67.41 (15.47,153.23)	150.89 (13.51,193.40)	0.106	34.84 (0.00,136.55)	153.91 (39.40,257.70)	0.006	130.77 (23.20,257.63)	150.18 (7.03,195.49)	0.590
Corin, Median (IQR) ng/mL	19.54 (4.92,27.00)	17.62 (5.22,24.26)	0.137	8.58 (3.58,27.26)	17.34 (4.42,23.78)	0.332	20.34 (5.65,27.47)	17.91 (7.22,24.87)	0.244

Abbreviations: BDNF, brain-derived neurotrophic factor; BNP, brain natriuretic peptide; NEP, neprilysin.

### Effect on short-term prognosis

**Fig 3:**
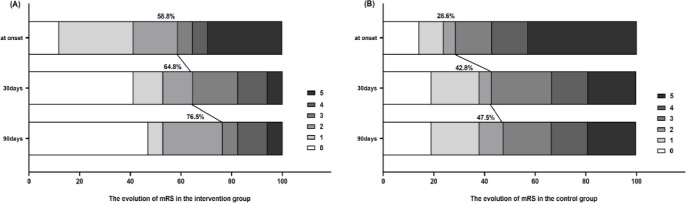
Evolution of mRS scores over time in the two groups.

Fig 3 shows the evolution of mRS scores over time in the two groups. At 30 days, 11 (64.8%) of the intervention group and 9 (42.8%) of the control group had a favorable functional outcome (mRS 0–2). Then at 90 days, 13 (76.5%) patients in the intervention group had a favorable functional outcome (mRS 0–2), and in the control group, 10 (47.5 percent) patients had a good functional outcome. Unfortunately, there was no statistically significant difference in functional prognosis between the two groups (all *P* values>0.05).

### Impact on NIHSS and mRS

**Fig 4:**
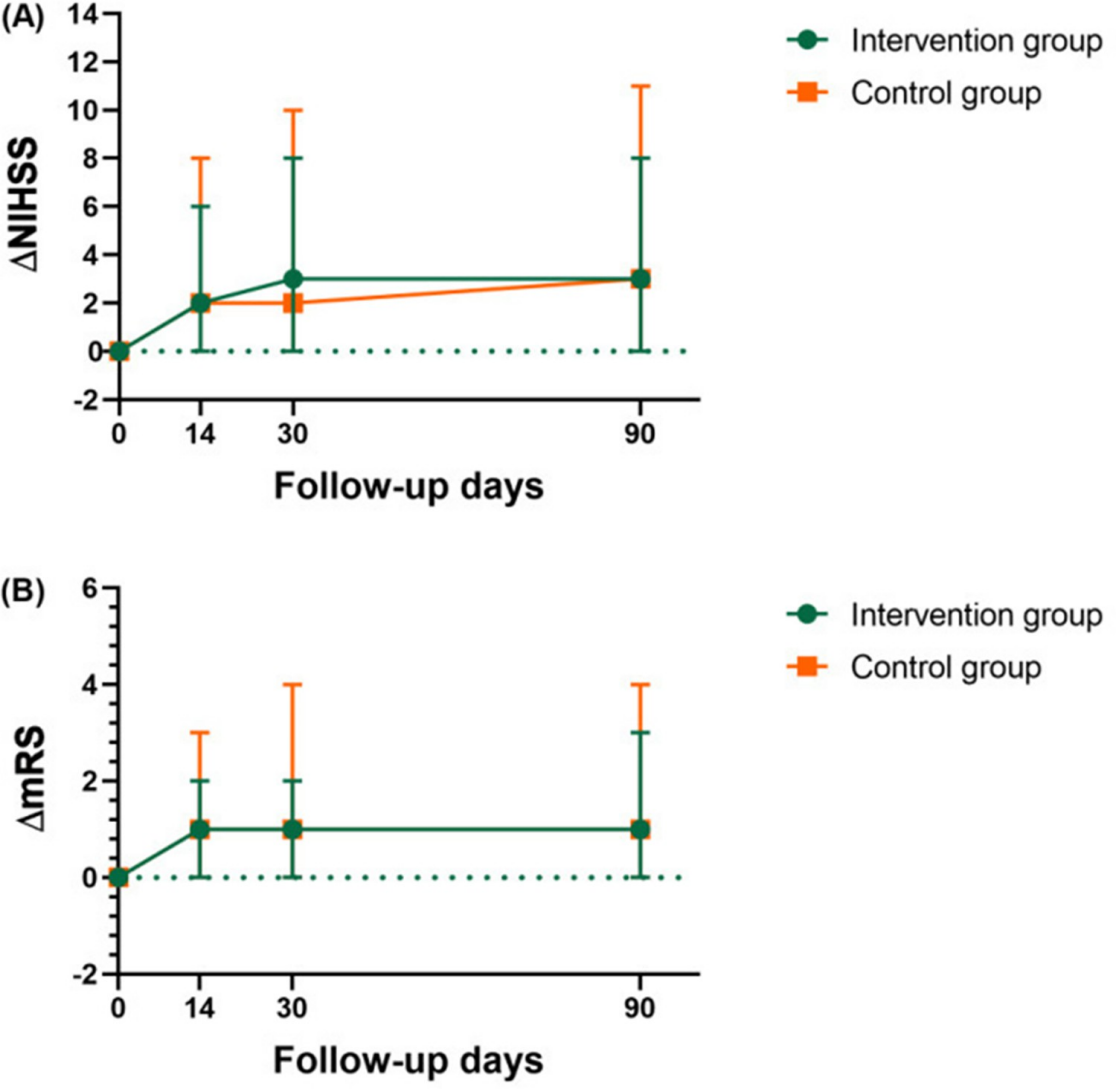
Evolution ofΔNIHSS andΔmRS over time in the two groups.

Mann-Whitney U test was used to analyze the effect of different interventions on mRS and NIHSS scores over time. The intervention group and control group of mRS scores had a declining trend over time, however, the trend did not differ statistically between the two groups (median difference mRS score from baseline to 14 days of follow-up, 1.0 vs 1.0, U = 159, *P* = 0.581; from baseline to 30 days of follow-up, 1.0 vs 1.0, U = 146, *P* = 0.352; from baseline to 90 days of follow-up, 1.0 vs 1.0, U = 142, *P* = 0.294). Similarly, NIHSS scores of the intervention group and the control group also showed a decreasing trend over time, and there was also no significant difference between the two groups (median difference NIHSS score from baseline to 14 days of follow-up, 2.0 vs 2.0, U = 172, *P* = 0.862; from baseline to 30 days of follow-up, 3.0 vs 2.0, U = 175, *P* = 0.931; from baseline to 90 days of follow-up, 3.0 vs 3.0, U = 174, *P* = 0.908) (Fig 4).

### Monitoring of blood pressure

**Fig 5:**
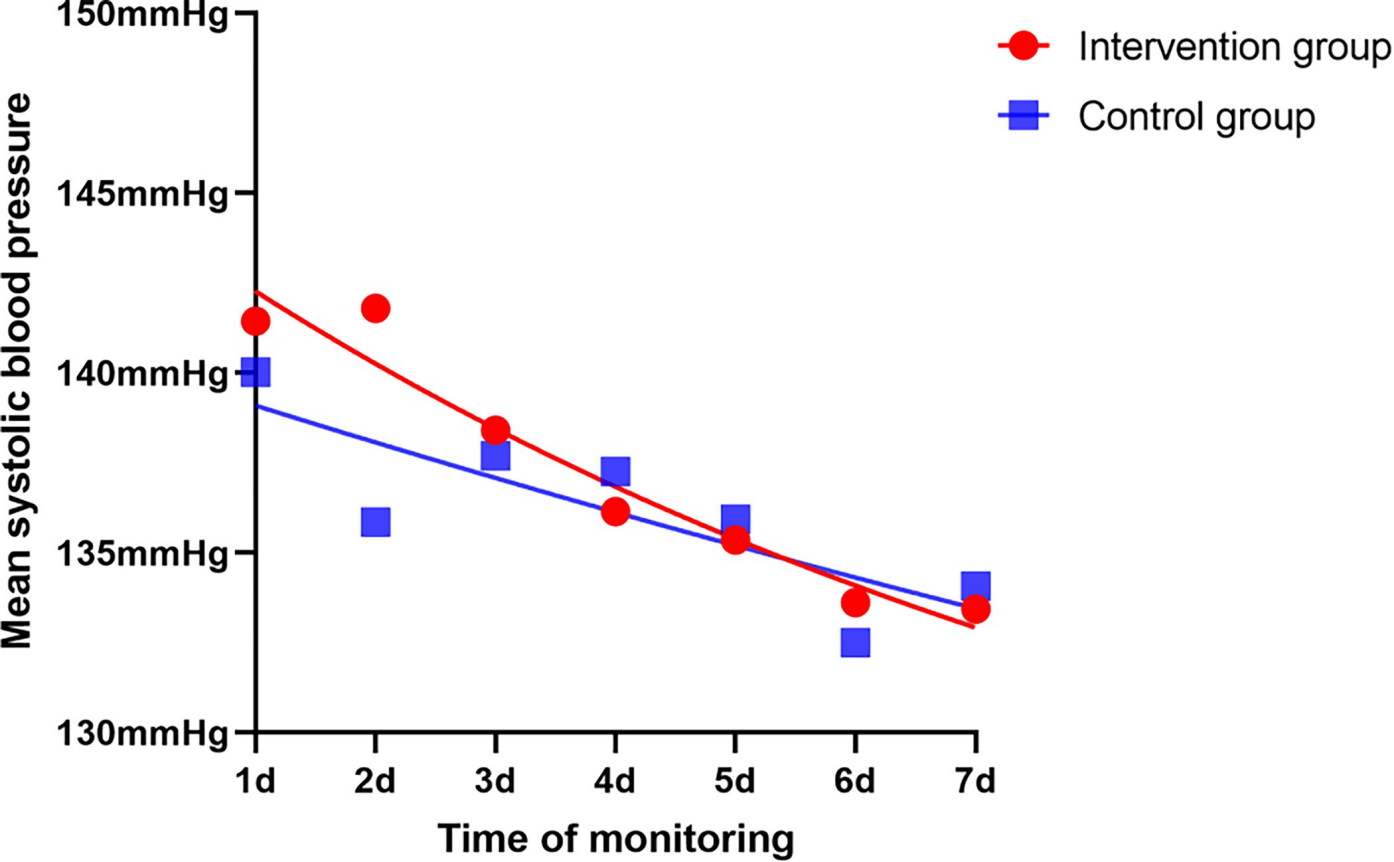
Monitoring of blood pressure.

**Fig 6:**
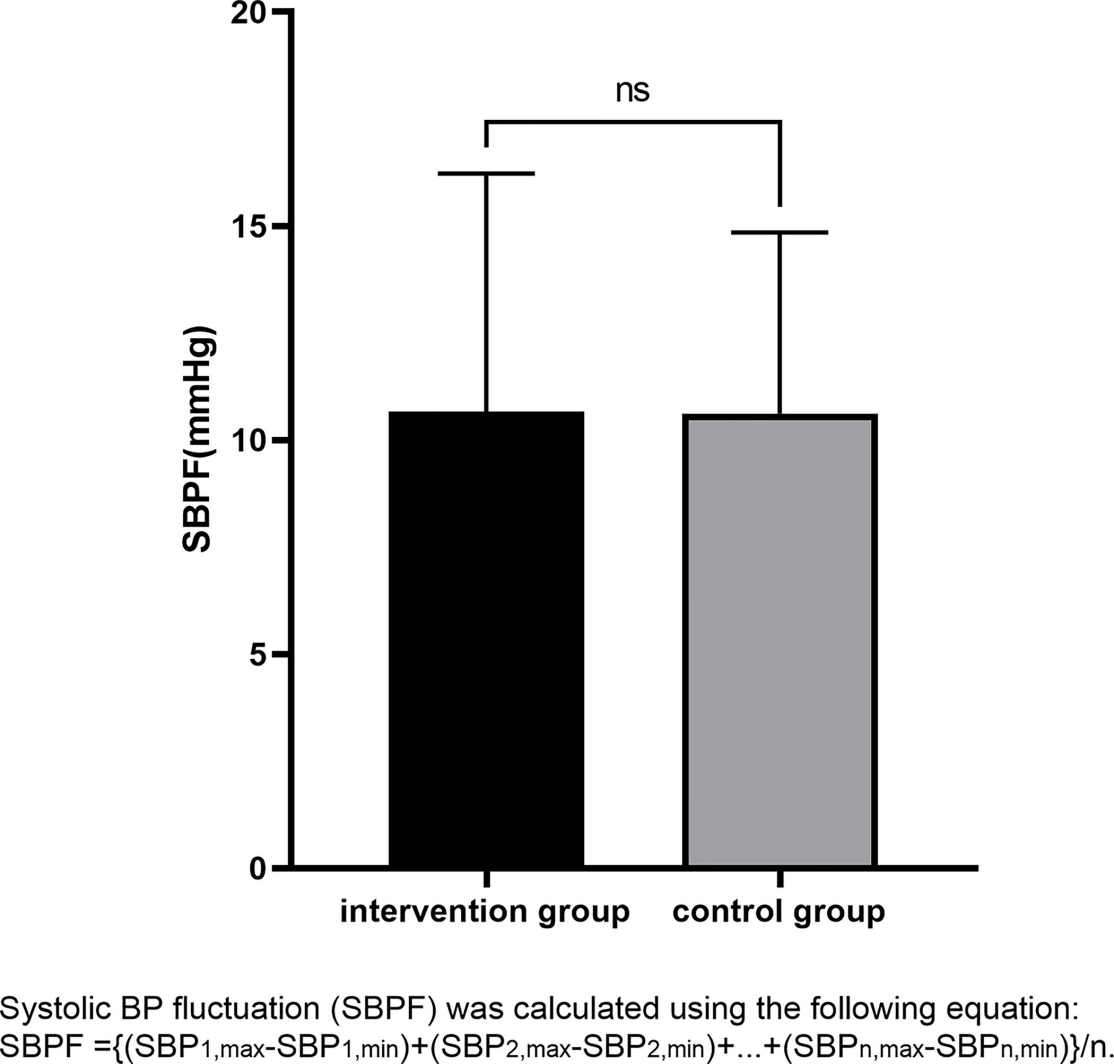
Fluctuations in systolic blood pressure.

As shown in Fig 5, the enrolled patients’ blood pressure were monitored during the week after the initiation of medication. Two-way repeated measures ANOVA was used to analyze the effect of different interventions on mean systolic blood pressure over time. The Mauchly’s test of sphericity for mean systolic BP of patients in two groups met the spherical symmetry hypothesis (*P*>0.05). The results showed that there was a statistical significance in mean systolic BP between the two groups at different time points (*P* = 0.001), but unfortunately there were no statistical significance between the two groups or interaction between groups and time (P>0.05) (Table 4). Furthermore, we analyzed the SBPF of the two groups, but also no statistically significant difference was found (Fig 6).

**Table 4:** ANOVA of mean systolic blood pressure in two groups.

Effects	Mean Square	F	P value
Between groups	63.390	0.057	0.813
Time	285.900	3.803	0.001
Interaction	53.628	0.713	0.639

### Adverse events and safety outcomes

The safety outcomes of the two groups during the trial could be seen in Fig 1. Two patients randomly assigned to the intervention group (to sacubitril/valsartan) occurred hypotension after medication during treatment; One patient assigned to the control group (to routine medicine) died of acute myocardial infarction during 90-day of follow-up. The main safety outcome, 90-day mortality, did not differ between the two groups. At the same time, due to the low incidence of adverse events, there was no difference between groups for any of the safety endpoints.

## Discussion

Changes in the hypothalamic-pituitary-adrenal (HPA) axis, the sympathetic nervous system, and the levels of several hormones, including natriuretic peptides, melatonin, and IGF-1, occur after a stroke [14]. Neuroendocrine activation after stroke and increased levels of natriuretic peptides could be an adaptive regulatory mechanism [15]. The potential mechanism of BNP elevation may have the following aspects. On the one hand, the increased intracranial pressure after an ACI, especially after a large cerebral infarction, causes mechanical compression of the hypothalamus, resulting in increased secretion of BNP [16]. On the other hand, after ACI, the blood pressure regulation mechanism may be activated reflexively, causing an increase in myocardial contractility and myocardial strain, which stimulates the release of BNP from cardiomyocytes [17]. In addition, brain tissue damage in ACI patients can cause dysregulation of the hindbrain-cardiac axis, stimulating increased production of cardiac-derived BNP, which can further cause cardiac damage [18]. The following are possible explanations for the underlying mechanisms of the hindbrain-cardiac axis: HPA axis activation, sympathetic and parasympathetic regulation, catecholamine surge, dysregulation of the gut microbiota, immune response, and release of inflammatory particles and microRNAs [19, 20].

BNP, Corin, and NEP are all important components of natriuretic peptides (NPs) [21, 22]. Corin-BNP-NEP constitutes a protein pathway from generation to decomposition [23]. BNP is cleaved from the proBNP precursor mainly by the enzyme Corin [24]. The decline in the enzyme Corin may play a role in the pathogenesis of decompensated congestive heart failure’s avid sodium retention, cardiac hypertrophy, and blunted brain natriuretic peptide actions [25]. In our study, there was no statistical difference in Corin changes, which we speculated might be due to the small sample size. Several enzymes are involved in NPs degradation, among which NEP plays a dominant role [26]. However, due to the large difference in baseline NEP levels before medication between the two groups in our study, the increase of NEP levels in the intervention group after the medication was more than that in the control group, which was of no significance for analysis.

LCZ696 (sacubitril/valsartan), the first of the new angiotensin receptor-neprilysin inhibitor (ARNI) drug class, contains equimolar amounts of sacubitril and valsartan [27]. Sacubitril is a drug that enters the body and is converted into the active NEP inhibitor LBQ657 [28]. Valsartan is an AT1R blocker that counteracts the Ang II elevation caused by enkephalins inhibitors [29]. Sacubitril/valsartan reduces readmission rates, signs, symptoms, and BNP levels at 4 weeks and 8 weeks of dosing in patients with acute heart failure [30, 31]. As reported in the PARADIGM-HF trial, circulating levels of BNP may increase meaningfully early after initiation of sacubitril/valsartan [32]. However, a decrease in plasma BNP levels was actually observed in the intervention group in our study. We try to analyze the reasons as follows: First, ANP may play a more important role due to different substrate specificity. In the natriuretic peptide family, NEP has high substrate specificity for both ANP and CNP, but low substrate specificity for BNP. This difference in substrate specificity can be attributed to their structural differences: Due to the short amino- and carboxy-terminal tails of ANP and the lack of carboxy-terminal tails of CNP lead to more efficient geometric interactions with the catalytic site and subsequent cleavage. In contrast, the long tails of BNP do not allow its ring to form appropriate substrate sites for catalysis, resulting in initial cleavage occurring outside the ring structure [33]. Ibrahim et al. [34] found that ANP concentrations were consistently and substantially increased after treatment with sacubitril/valsartan, while BNP concentrations differed across measurements, and CNP circulating concentrations were lower and unaffected by NEP inhibition. This is consistent with our conjecture that the benefit of NEP inhibitors may be better explained by an increase in ANP concentration. Second, In addition to NPs, other NEP substrates (e.g. adrenomedullin, bradykinin, urodilatin, substance P, vasoactive intestinal peptide, calcitonin gene-related peptide, glucagon-like peptide-1) may also play a major role in the mechanism of action of sacubitril/valsartan [35, 36], which needs to be confirmed by further studies in the future. Third, levels of the N-terminal prohormone of BNP (NT-proBNP) and troponin were significantly lowered in patients treated with sacubitril/valsartan, reflecting a reduction in myocardial wall stress and cardiac damage, respectively. These changes, in turn, will lead to a corresponding reduction in the stimulus generated by BNP [36, 37]. Finally, since various BNP immunoassays are based on several combinations of captured and detected antibodies that detect different BNP degradation products, the results of each assay may vary [34]. In conclusion, we suggest that sacubitril/valsartan may effectively reduce myocardial wall stress mainly by increasing plasma ANP concentration or activating other NEP substrates, ultimately leading to a reduction in BNP production stimulation, which should be sufficient to offset the increase in plasma BNP caused by inhibiting NEP. It is worth mentioning that in our study, similar doses of valsartan were selected instead of placebo as the control group, in order to minimize the effect of the AT1R blocker on the RAAS system and thus reduce the impact on the final results.

Elevated natriuretic peptide levels are associated with worse clinical outcomes in stroke, which might demonstrate the relatively low level of protection brought on by this endogenous effect [38]. As a result, the natriuretic peptide system is markedly elevated after stroke, and this elevation is more likely a regulatory adaptation than a harmful consequence [39]. Our trial intervened with sacubitril/valsartan in patients with ACI to observe whether the intervention on natriuretic peptide level affects stroke prognosis.

BNP levels are valuable predictors of stroke outcome [40]. A study of 441 CE patients > 80-year-old with 90-day and 1-year follow-up demonstrated that BNP, but not s-cTnI, was an independent predictor of death [41]. In addition to the prognostic value of stroke, the natriuretic peptide system itself may have a protective effect on the brain, heart, and kidneys [42, 43]. Sacubitril/valsartan inhibits the RAAS system while inhibiting enkephalinase, which enhances the target organ protection of endogenous natriuretic peptides [44]. These protective effects are primarily achieved through natriuretic and diuretic agents, vasodilators, RAAS and sympathetic activity inhibition, weak cardiac remodeling and fibrosis, reversal of vascular remodeling (atherosclerosis), reduction of renal fibrosis, improvement of renal hemodynamics, increase in endothelial function, lipid mobilization, etc [45]. No difference has been observed in functional prognosis between the two groups in this study, which does not provide evidence for the neuroprotective effect of sacubitril/valsartan. But it may be because this study did not include Imaging indicators such as cerebral blood perfusion, brain tissue metabolism, and changes in infarct volume were used to reflect the effectiveness of intervention studies. Besides, increased variability in blood pressure will significantly increase the risk of stroke recurrence, major cardiovascular events, and death. Considering that most of the patients enrolled in our trial had a history of hypertension for many years and were regularly taking anti-hypertensive drugs prior to admission, we did not select placebo as the control group, which we believe was one of the main reasons why there was no significant difference in prognosis between the two groups.

BDNF plays an important role in neuroprotection and is involved in the anti-hypoxic/ischemic injury pathway [46–48]. BDNF is used to evaluate the effects of intervention factors in prospective interventional studies [49, 50]. According to a prospective cohort study of sICH revealed by microarray analysis and protein functional enrichment analysis, BDNF is a neuroprotective biomarker associated with brain injury in sICH after thrombolysis [51]. Studies have shown that aerobic exercise after ischemia/reperfusion may promote neuroprotective mechanisms and neuronal repair and survival that are partially mediated by BDNF [52]. No difference was seen in plasma BDNF levels between the experimental and control groups in this study. Sacubitril/valsartan’s neuroprotective mechanism has not been established, and it may promote euro-protection via another pathway.

### Advantages

The experiment is a prospective study, the research factors are pre-designed, the outcome variables and measurement methods are pre-specified, and the final argument is strong by comparing with the control group. It has important clinical significance for the safety and efficacy of sacubitril/valsartan in the treatment of ACI.

### Limitations

This was a single-center study, and the sample size was not large enough. Perhaps the results of expanding the trial will be more applicable to our clinical practice. In addition, it is unfortunate that plasma ANP and CNP levels were not measured in this study due to due to their rapid clearance in circulation. Imaging indicators such as cerebral blood perfusion, brain tissue metabolism, and changes in infarct volume were not used as efficacy evaluation indicators. Plasma BNP, Corin, and NEP levels were tested less frequently, only before and 5 days after administration.

## Conclusions

Sacubitril/valsartan reduced plasma BNP levels in patients with ACI and did not affect their short-term prognosis.

## Supporting information

S1 Checklist CONSORT 2010 checklist of information to include when reporting a randomised trial*. (DOC)

S1 File Experimental study protocol. (DOCX)
