# Supplementary material for: Effect of sacubitril/valsartan on brain natriuretic peptide level and prognosis of acute cerebral infarction
Source: PLoS One. 2023 Sep 21;18(9):e0291754. doi: 10.1371/journal.pone.0291754 (PMC10513241; doi:10.1371/journal.pone.0291754)
Supplement: S1 File — (DOCX) [file pone.0291754.s002.docx]

**Experimental Study Protocol**

**1.The name of the research project：**

Effect of sacubitril/valsartan on brain natriuretic peptide level and prognosis of acute cerebral infarction

1. **The objectives of the research project：**

Previous studies demonstrated that elevated brain natriuretic peptide (BNP) level is associated with adverse clinical outcomes of acute cerebral infarction (ACI). Researchers hypothesized that BNP might be a potential neuroprotective factor against cerebral ischemia because of the antagonistic effect of the natriuretic peptide system on the renin-angiotensin system and regulation of cardiovascular homeostasis. However, whether decreasing the BNP level can improve the prognosis of ACI has not been studied yet. The main effect of sacubitril/valsartan is to enhance the natriuretic peptide system.

The aim of the research is to explore whether the intervention of plasma BNP levels with sacubitril/valsartan could improve the prognosis of patients with ACI.

1. **Main problems to be solved:**

Valify at the clinical level that intervention natriuretic peptide levels may improve the prognosis of patients with ACI.

1. **Research ideas and methods:**
2. 80 patients aged≥18 years who had ACI confirmed by computed tomography (CT) or magnetic resonance imaging (MRI) of the brain within 48 hours of symptom onset and required antihypertensive therapy (Real-time systolic BP ≥180mmHg or diastolic BP≥100mmHg, or patients with severe cardiac insufficiency, aortic dissection, hypertensive encephalopathy, or having a history of hypertension and regularly taking anti-hypertensive medication) were consecutively enrolled. All subjects were required to sign an informed consent form.

(B) Patients who fulfilled one of the following conditions were excluded from the trial: (1) Patients with cerebral hemorrhage and occupancy (emergency head CT excludes cerebral hemorrhage, while post-infarction hemorrhage is not excluded if the patient was hospitalized); (2) Patients with transient ischemic attack; (3) Complicated with severe infection or septic shock; (4) Combined with hypotension or hyperkalemia; (5) Obvious hepatic and renal insufficiency (glomerular filtration rate <30ml/min or Child-Pugh class C); (6) Endocrine, immune, or neoplastic diseases; (7) History of severe trauma with surgical treatment within 30 days; (8) Presence of blindness, deafness or communication disorders; (9) Diagnosed as schizophrenia, affective disorder, organic mental disorder or mental retardation; (10) had a contraindication to sacubitril/valsartan; (11) Pregnancy.

(C) Exit criteria: 1) The patient is unwilling to continue to cooperate with the treatment; 2) Lost to follow-up or incomplete patient data during follow-up; 3) Hypotension occurs during the medication process.

(D) Intervention methods: Participants were allocated in a 1:1 ratio to receive either sacubitril/valsartan treatment (intervention group) or conventional medical therapy (control group). The randomization sequence was computer-generated. A specialist who was not involved in the trial performed the randomization process. Sacubitril/valsartan 200mg once daily or conventional medical drugs (Valsartan 160mg once daily) were given as soon as possible within 24 hours of randomization, and other post-admission treatment regimens remained as consistent as possible. Over the course of more than 3 months, sacubitril/valsartan has been given. 5 ml of peripheral venous blood was provided before and after 5 days of medication. Blood samples were collected for pretreatment in the laboratory, serum and blood cells were separated, stored in a refrigerator at -80℃, and transported for examination.

(E) Observation indicators: 1) biochemical indicators: before and after medication, 5ml random venous blood was used to detect brain-derived neurotrophic factor (BDNF), CRN, BNP and NEP; 2) Blood pressure monitoring of the enrolled patients during hospitalization.

(F)The follow-up plan: The mRS and NIHSS scales were completed by in-hospital interview or telephone interview at the time of onset, discharge, 1 month and 3 months.

(G) Statistical analysis: All statistical analyses were performed using SPSS26.0 software. Chi-square test was used to compare categorical variables and Kolmogorov-Smirnov Z test was used to compare continuous variables. All statistical tests were two-sided and a P＜0.05 was considered significant.

1. **Innovation:**

The possibility of changing the level of natriuretic peptide to affect the short-term prognosis of patients with ACI was investigated for the first time in clinical trials in China.

**6. Research basis and working conditions:**

Lianyungang Second People’s Hospital is a tertiary general hospital integrating medical treatment, teaching and research in Lianyungang area. Geriatric medicine is a first-class key specialty at the municipal level. Relying on the laboratory of medical laboratory, it has undertaken many provincial and municipal scientific research projects and successfully concluded the projects.

**7.Cost estimates：**

| Item of expenditure | Amount (ten thousand yuan) | Specific projects and reasons |
| --- | --- | --- |
| Scientific research | 0.5 | Research, academic exchange, novelty search, published papers, etc |
| Expendable experimental materials | 1.0 | All kinds of experimental supplies |
| Instrument and equipment usage fee | 0.5 | Equipment needed for the experiment |
| Total | 2.0 |  |
